# Retraction Note: Fatal factitious Cushing syndrome (Münchhausen’s syndrome) in a patient with macroprolactinoma and silent corticotrophinoma: case report and literature review

**DOI:** 10.1186/s40842-016-0022-z

**Published:** 2016-03-03

**Authors:** Carlos André Minanni, Ana Luiza De Almeida Cardoso, Edoarda Vasco de Albuquerque Albuquerque, Luciana Pinto Brito, Ludmilla Malveira Lima Lopes, Andrea Glezer, Elisa Del Rosario Ugarte Verduguez, Berenice Bilharinho De Mendonça, Marcello Delano Bronstein, Marcio Carlos Machado, Maria Candida Barisson Villares Fragoso

**Affiliations:** 1 Neuroendocrinology Unit, São Paulo, Brazil; 2 Laboratory of Hormones and Molecular Genetics – LIM/42, São Paulo, Brazil; 3 Division of Psychology, Adrenal Unit, São Paulo, Brazil; 4 Hospital das Clinicas da Faculdade de Medicina, University of São Paulo, São Paulo, Brazil

The Publisher is retracting this case report and literature review [1] as the case has been previously published in the *Journal of Women’s Health Care* [2] and is therefore a redundant publication.

## References

[1] Minanni CA, De Almeida Cardoso AL, de Albuquerque Albuquerque EV, Pinto Brito L, Lima Lopes LM, Glezer A, Del Rosario Ugarte Verduguez E, Bilharinho De Mendonça B, Bronstein MD, Machado MC, Fragoso MCBV. Fatal factitious Cushing syndrome (Münchhausen’s syndrome) in a patient with macroprolactinoma and silent corticotrophinoma: case report and literature review. Clin Diabetes Endocrinol. 2015;1:3. 10.1186/s40842-015-0002-8 PMC5469202 28702222

[2] Minanni CA, Cardoso ALA, Albuquerque EVA, Lopes LML, Glezer A, Verduguez ERU, Gallucci-neto J, Gattaz WF, Mendonça BB, Bronstein MD, Machado MC, Fragoso MCBV. Fatal factitious Cushing’s syndrome (Münchhausen’s syndrome) in a patient with a prolactinoma and silent corticotrophinoma: case report and literature review. J Women’s Health Care. 2014;3:162.

